# Caveat Pre-Emptor: Contextualising peer review and publication

**DOI:** 10.1371/journal.pbio.3000234

**Published:** 2019-05-22

**Authors:** The PLOS Biology Staff Editors

**Affiliations:** Public Library of Science, San Francisco, California, United States of America and Cambridge, United Kingdom

## Abstract

This Editorial describes PLOS Biology's experiment with contextualised publication, whereby potentially controversial studies are published with an Editor’s Note and an accompanying Primer in which the Academic Editor guides the reader, dissects the study, and lays out the thinking behind the decision to publish.

PLOS journals are today launching optional published peer review, in which authors can elect to publish, alongside their papers, the decision letters, reviewer comments, and their own responses. We at *PLOS Biology* feel this is great news; opening up peer review is a natural progression in the movement that recognizes both the utility and moral mandate for open access publication and for making both data and code as well as unique research materials available. Publishing the reviews will provide important context for decisions and help others interpret the research. For a small subset of papers, however, we’re finding that the route to publication can benefit from some very special additional treatment. For these particular manuscripts, it is clear that the claims are potentially intriguing and important, but the reviewers’ comments indicate that simple publication might lead to misunderstanding or misinterpretation. We believe that such cases benefit from contextualised publication with an Editor’s Note on the article and an accompanying Primer in which the Academic Editor carefully guides the reader, dissects the study, and lays out the thinking behind the decision to publish.

## Contextualised Publication: Explaining our decision to publish

Even if we don’t advertise them widely, as former scientists we at *PLOS Biology* always like to run experiments, taking opportunities to push the boundaries of openness. Our editorial model is already quite unique and consists of discussing a submitted manuscript with an Academic Editor, seeking the opinions of several expert reviewers, and then discussing the reviewers’ comments with the Academic Editor as we formulate a decision. To recognise this effort, the Academic Editor’s name is published within any accepted manuscripts as an imprimatur.

Our experience as editors has taught us that for the vast majority of manuscripts, this model tends to converge (perhaps after revisions) on a relatively straightforward decision—to publish, to invite revision, or to reject. But what if this resolution doesn’t happen? What if, after one or two rounds of review, there are still deep divisions between the opinions of conscientious and constructive reviewers, or individual reviewers are themselves in two minds as to whether to recommend publication? At such an impasse, the problems are exacerbated if the claims of the paper concerned are potentially important and contentious for the field.

Simply publishing such a paper as-is might run the risk of mis- or over-interpretation. On the other hand, rejecting it would not only be potentially unfair to the authors, but would also render unpredictable how that paper might eventually be published and promoted. Moreover, the substantial time and effort already spent by multiple well-qualified academics in reviewing the manuscript and providing their expert opinions might go to waste. And crucially, by publishing the study, we have the opportunity to work with the authors to determine how the results of a study are communicated, as opposed to leaving it to the vagaries of publication elsewhere.

**Fig 1:**
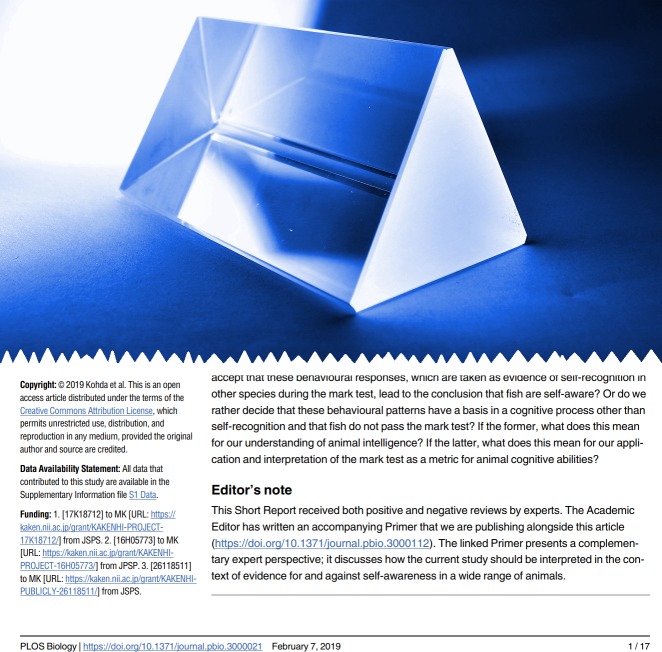
In contextualised publication, the Academic Editor’s Primer is envisaged as the prism through which the research paper should be viewed. The lower panel shows the Editor’s Note from one of the papers described in Box 1. *Image credit*: Anna Miska-Alvarez and https://doi.org/10.1371/journal.pbio.3000021.

This has happened to us three times in the past year (see Box 1), and it has allowed us to hone a process to deal with this eventuality, which we’d like to share. All three cases had their idiosyncrasies, but the outcome in each instance was the same. Rather than publish the paper in isolation or reject it, we reached an agreement with the Academic Editor and the authors regarding mutually satisfactory terms of publication: namely, co-publication with a linked Primer written by the Academic Editor, in which she or he provides context to the study, discusses any perceived or real limitations (perhaps paraphrasing the reviewers’ concerns), explains the rationale behind the decision to publish, and outlines further work that is needed to resolve any remaining questions. In each case the “unit of publication” was the research article plus the Primer, the former bearing a special Editor’s Note advising readers that the two should be read in conjunction (Fig 1). In short, we were able to publish these intriguing and thought-provoking studies while contextualising the background behind the decision to publish—the only piece missing for these papers were the peer reviews, and of course going forwards, if the authors agree, these too will be available.

## And in the future?

In the three cases that we describe here, the need for contextualisation became gradually apparent during the process. We’ve now formalised how we might identify such cases in the future and devised a recommended workflow, to try to ensure that this occurs in a more structured way. By doing this, we hope to be able to publish more provocative science in a more contextualised environment, giving readers a more nuanced understanding of the research as well as its limitations and promise. The initiative seems to have largely been well received by readers, reviewers and authors. And once reviews are made openly available, readers will be privy to the underlying concerns that drove our decisions and this particular mode of publication.

**Box 1. Examples of contextualised publication**

Below are three instances in which a research paper has been published on the condition of co-publication with a Primer specially commissioned to contextualise the work, to explain the decision to publish, and to caveat some of the conclusions.

**A. Hot Mitochondria**

What the paper said:

Results obtained from a mitochondrion-targeted temperature-sensitive fluorescent probe suggest that the internal mitochondrial temperature is substantially higher (by 10°C) than the surrounding intracellular space [1]. Mitochondrial enzymes seem to be optimised to this higher temperature.

What the Primer said:

Nick Lane: While these conclusions are surprising, readers should bear in mind that other factors than temperature might be affecting the probe, and that the implied temperature gradient across the mitochondrial membrane is hard to explain. That said, mitochondria do produce substantial amounts of heat, and the study raises interesting questions about the meaning of “temperature” in tiny volumes and non-equilibrium turbulent conditions [2].

**B. Electromagnetic field effect on cells**

What the paper said:

Studies using fruit flies and cultured mammalian cells suggest that low-intensity electromagnetic fields can influence biological systems by triggering the production of reactive oxygen species (ROS) via a mechanism that is dependent on cryptochromes, proteins implicated in magnetoreception [3].

What the Primer said:

Lukas Landler and David Keays: Previous studies in this field have been justifiably viewed with scepticism, and the topic lies at the heart of a controversy about alleged adverse health effects of power lines. The current paper represents a substantial improvement, but the implementation of zero magnetic field controls remains problematical, and the role of cryptochromes may be indirect [4].

**C. Fish in a mirror**

What the paper said:

Experiments using cleaner wrasse suggest that they are able to identify their reflection in a mirror as themselves, and will attempt to remove marks only visible in the mirror by scraping their bodies on stones. This suggests that these fish exhibit a level of self-awareness that has only been demonstrated in a handful of species [5].

What the Primer said:

Frans de Waal: These findings are intriguing, but there remains a question about the control invisible mark, which may have a tactile component, and about what this so-called “mark test” really tells us about animals’ sense of self. The study supports the case for a more gradualist approach to animal self-consciousness [6].
